# Associations between Body Mass Index and Subjective Health Outcomes among Older Adults: Findings from the Yilan Study, Taiwan

**DOI:** 10.3390/ijerph15122645

**Published:** 2018-11-26

**Authors:** Hsiao-Ting Chang, Nai-Wei Hsu, Hsi-Chung Chen, Hsuan-Ming Tsao, Su-Shun Lo, Pesus Chou

**Affiliations:** 1 Department of Family Medicine, Taipei Veterans General Hospital, Taipei 11217, Taiwan; htchang.tw@gmail.com; 2 School of Medicine, National Yang-Ming University, Taipei 11221, Taiwan; davidnwh@ms28.hinet.net (N.-W.H.); hmtsao@ymuh.ym.edu.tw (H.-M.T.); sslo@ymuh.ym.edu.tw (S.-S.L.); 3 Division of Cardiology, Department of Internal Medicine, National Yang-Ming University Hospital, Yilan 26042, Taiwan; 4 Department of Psychiatry & Center of Sleep Disorders, National Taiwan University Hospital, Taipei 10048, Taiwan; hsichungchen@ntu.edu.tw; 5 Department of Surgery, National Yang-Ming University Hospital, Yilan 26042, Taiwan; 6 Community Medicine Research Center and Institute of Public Health, National Yang-Ming University, Taipei 11221, Taiwan

**Keywords:** body mass index, older adults, health-related quality of life, self-rated health, Self-rated happiness

## Abstract

Previous findings on the associations between body mass index (BMI) and subjective health outcomes among older adults are inconsistent. The aims of this study were to explore the associations of BMI with health-related quality of life (HRQoL), self-rated health (SRH) and happiness among older adults. This study was part of the Yilan study, which was a community-based survey conducted in the Yilan city in Taiwan. A total of 3722 older adults were randomly recruited during 2012–2016. HRQoL was measured using the Short Form-12 Health Survey physical component summary (PCS) and mental component summary (MCS) scores and SRH and happiness were also evaluated. By hierarchical regression, after adjusting for covariates, compared with normal-weight participants, overweight did not have significantly different PCS scores (B = 0.20, 95% confidence interval [CI]: −0.45 to 0.85, *p* = 0.546) but obese had significantly lower PCS scores (B = −0.97, 95% CI: −1.68 to −0.26, *p* < 0.0001); overweight and obese participants had significantly better MCS scores (B = 1.00, 95% CI: 0.40 to 1.61, *p* = 0.001 and B = 1.22, 95% CI: 0.60 to 1.88, *p* < 0.0001, respectively); overweight participants had significantly higher SRH scores (B = 1.08, 95% CI: 0.16 to 2.00, *p* = 0.022) but underweight had significantly lower SRH scores (B = −2.88, 95% CI: −4.81 to −0.95, *p* = 0.003); overweight and obese participants had better happiness scores (B = 1.55, 95% CI: 0.45 to 2.66, *p* = 0.006 and B = 1.68, 95% CI: 0.49 to 2.88, *p* = 0.006, respectively). In conclusion, compared with normal-weight individuals, overweight individuals had better mental HRQoL, SRH and happiness but underweight older people reported poorer SRH and obese reported poorer physical HRQOL but better mental HRQoL and self-rated happiness.

## 1. Introduction

According to the World Health Organization’s definition of health, health is not merely the absence of disease or infirmity; subjective feelings of physical, mental and social health or well-being are also important [1]. Besides, subjective health outcomes are also found to be related to important objective health outcomes. People with poorer health-related quality of life or self-rated health experience a higher risk of morbidity and mortality [2,3,4]. Therefore, many measurements have been developed and used to evaluate subjective health outcomes. The Short Form-12 Health Survey, Version 2 (SF-12 v2) is widely used to assess health-related quality of life (HRQoL) [5,6,7,8]. A single question (“How would you rate your present health status?”) is often used to gauge self-rated health, either providing set response categories [2,3] or using a 100-point visual analogue scale [9]. Happiness is another outcome variable used to evaluate quality of life as a whole. In the United States General Social Survey, happiness is operationalized as a single question with set response categories and this question has been repeated in several waves of this survey [9,10]. Thus, the SF-12, self-rated health and happiness are measures used to evaluate different aspects of subjective health.

Body mass index (BMI) is a measurement commonly used in research to evaluate subjects’ body composition status. Previous studies that focused on associations between BMI and health outcomes have found that being underweight, overweight, or obese is associated with adverse health outcomes. Overweight and obesity are risk factors for metabolic syndrome, type 2 diabetes, cardiovascular diseases, cerebrovascular diseases, certain cancers and mortality [11,12,13,14,15]. However, studies focusing on the associations between BMI and subjective health outcomes have shown inconsistent results: some have found that being overweight or obese is associated with poorer quality of life [16,17,18,19,20,21], self-rated health [4,22] and well-being [18], whereas others have found that older overweight adults experience better subjective health outcomes than do their normal-weight counterparts [19,4,23,24]. As for the associations between being underweight and health outcomes, many studies have found that underweight is related to increased morbidity, including frailty, disability, chronic conditions and sarcopenia, as well as to increased mortality among older adults [11,13,14,25,26,27,28]. Being underweight has also been shown to be related to worse HRQoL [17,20,29,30]. Several cross-sectional studies have shown that being underweight is associated with poor physical and mental health [17,23,30] whereas other investigations have found only the association with poor mental health [20]. One 2-year follow-up study conducted in England which included people ≥ 52 years old found that BMI had a protective effect on quality of life measured using CASP-19 (Control, Autonomy, Self-Realization and Pleasure) for women [31]. Another 4-year follow-up study in Brazil which investigated adults ≥ 60 years old found that maintaining a BMI within normal limits was helpful in preserving CASP-19 scores [32]. Still another 2-year follow-up study in Australia which included subjects 70–90 years old at baseline found that BMI had negative effects on independent living, social relationships and the experience of pain [33]. 

Subjective health outcomes are important aspects of care for older people. These outcomes reflect people’s sense of health and their capacity to react to various factors in their lives [1,17,34,35,36,37,38,39]. Previous findings on the associations between BMI and HRQoL among older adults are inconsistent in cross-sectional and longitudinal studies and studies on the associations of BMI with self-rated health and happiness are scant. Therefore, the aim of the present study was to examine the associations between BMI categories and subjective health outcomes, including HRQoL, self-rated health and self-rated happiness among older adults living in the community. The results of this study would provide information for ideal body weight for older adults to maintain better physical and mental HRQOL, self-rated health and self-rated happiness.

## 2. Materials and Methods

### 2.1. Setting and Subjects

This population-based community health survey was conducted by the Community Medicine Research Center of National Yang-Ming University and National Yang-Ming University Hospital in Taiwan. This study was conducted in Yilan, a moderately urban city in northeastern Taiwan. The inclusion criteria for participation were local residents who aged ≥ 65 years old who agreed to participate. Those who were living in the long-term care facilities, those who were unable to complete the anthropometric measurements including those had pace maker implementation, those had nails or screws inserted due to previous bone fractures (for safety reason, because the body weight was measured by electric body composition monitor) and those who were unable to complete the questionnaire were excluded. Potentially eligible subjects were identified and asked to participate randomly by well-trained study assistants door by door.

### 2.2. Measurements

#### 2.2.1. Demographic Characteristics

The survey recorded respondents’ age (categorized as 65–74, 75–84, or ≥ 85 years), sex, height (m), weight (kg), BMI (kg/m^2^, categorized as underweight [<18.5], normal weight [18.5–23.9], overweight [24.0–26.9], or obese [≥27], according to the BMI category defined by the Health Promotion Administration, Taiwan), educational level (illiterate, literate/elementary school, junior/senior high school and university or above), living status (living alone or living with others), cigarette smoking (nonsmoker, current smoker, or former smoker), alcohol consumption (nondrinker, current drinker, or former drinker) and community volunteer activities in the last month (yes or no).

#### 2.2.2. Anthropometrical Assessments

The height was measured by measuring tape and the weight was measured by Tanita Inner Scan Body Composition Monitor for one time but if the participants did not agree with the results, another measurement was done to check the results again. The anthropometrical assessments were performed by the same study assistant. 

#### 2.2.3. Medical Conditions, HRQoL, Self-rated Health and Self-rated Happiness

Self-reported disease status and treatment for medical conditions including hypertension (yes/no), diabetes mellitus (yes/no), cardiovascular diseases (yes/no) and stroke (yes/no) were recorded. HRQoL was measured using the Chinese version of the SF-12 v2, which is a shorter version of the Short Form-36, Version 2. [5,6,7,8] SF-12 v2 scores were grouped into physical component summary (PCS) and mental component summary (MCS) scores, indicating subjective physical health and mental health, respectively. Self-rated health was measured by asking “How would you rate your present health status?” [9] and self-rated happiness was measured by asking a single question: “How happy are you currently?” Responses to both questions were rated on a 0–100 scale. Higher scores indicated better HRQoL, better self-rated health and better self-rated happiness. 

### 2.3. Procedure

#### 2.3.1. Ethical Statement

The institutional review board of National Yang-Ming University Hospital in Taiwan approved this study and the informed consent forms and all participants provided written informed consent before participation (IRB Approval No.: 2011A016).

#### 2.3.2. Data Collection 

Face-to-face interviews were conducted by well-trained study assistants at the participants’ homes or at health stations to complete the measurements.

### 2.4. Statistical Analysis

**Figure 1:**
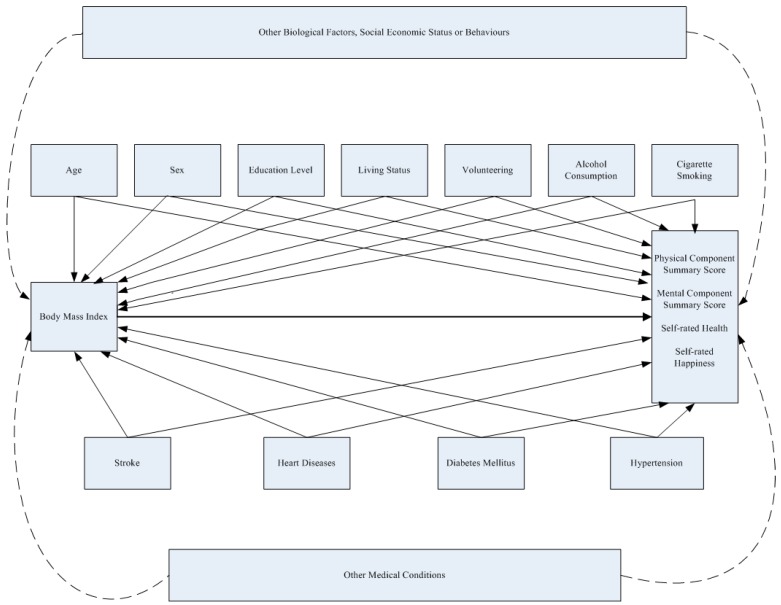
The Directed Acyclic Graph for the Associations between BMI, Covariates and the Study Outcomes.

Statistical analyses were performed using IBM SPSS, Version 20.0 (IBM Corporation, Armonk, NY, USA). Demographic characteristics were compared between males and females using Chi-squared tests; linear trends of the rate of medical conditions in BMI categories were analyzed by linear by linear association using Chi-squared tests. Scores of PCS, MCS, self-rated health and self-rated happiness between BMI categories were analyzed by ANOVA under the assumption of independence, normal distribution of these scores and with similar variance across BMI categories and Bonferroni adjustments were applied for post-hoc analysis. Unadjusted analysis of associations between variables and scores of PCS, MCS, self-rated health and self-rated happiness were analyzed by simple linear regression for each variable. Hierarchical regression models with all variables entered simultaneously were used to measure the associations of BMI categories with PCS, MCS, self-rated health and self-rated happiness scores, with adjustment for covariates (age, sex, educational level, living status, smoking, alcohol consumption, volunteering and the chronic conditions of hypertension, heart disease, diabetes mellitus and stroke) under the assumption of linear relationships between BMI and subjective health outcome scores with normal distributed residuals. Categorical variables including BMI, education level, age, cigarette smoking and alcohol consumption were classified into dummy variables with the reference group as the BMI of normal weight, education level of illiterate, age of 65–74 years, nonsmoker and nondrinker, respectively. The assumption of normally distributed residuals was checked in each model by using the Kolmogorov-Smirnov test. Multicollinearity was tested by eigenvalue and condition index; Cook’s distances were tested for outliers and constancy of variance of each subjective health outcome for covariates were checked by plots of Studentized residuals versus predicted values. A two-tailed *p*-value of <0.05 was considered statistically significant. The directed acyclic graph (Figure 1) was drawn to help to clarify the possible associations between BMI, covariates and the study outcomes. Age, sex, education level, living status, volunteering, alcohol consumption and cigarette smoking were biological factors, demographic characteristics and behaviors which were found to be associated with BMI and subjective health outcomes. Medical conditions included stroke, heart diseases, diabetes mellitus and hypertension were also found to be associated with BMI and subjective health outcomes in previous studies [5,6]. The associations of unmeasured potential confounders of biological factors, social economic status, behaviors and medical conditions between BMI and study outcomes were presented with dashed lines. 

## 3. Results

### 3.1. Participants’ Demographic Characteristics

A total of 3722 participants were included in this study with the mean ± SD age of 76.2 ± 6.5 years old. Among them, 1611 (43.4%) were male, 1912 (51.4%) were aged 65–74 years, 1605 (43.1%) had an educational level of literate/elementary school, 3445 (92.0%) lived with others, 2826 (75.9%) were nonsmokers, 3031 (81.4%) were nondrinkers and 432 (11.6%) had worked as volunteers. The mean BMI was 24.7 ± 3.8 kg/m^2^ and 1500 (40.3%) of the participants had a BMI between 18.5 and 23.9 kg/m^2^ (normal weight). Women in the study were younger, more likely to be obese, less educated, more likely to live alone and less likely to smoke or drink compared to men. There was no evidence that volunteering behavior differed between men and women (Table 1).

### 3.2. Participants’ Medical Conditions by BMI Category

A total of 2086 (56.0%) participants had hypertension, 866 (23.3%) had diabetes mellitus, 1131 (30.4%) had cardiovascular diseases and 167 (4.5%) had experienced a stroke. As BMI increased, there were significant increasing linear trends in the rates of hypertension (38.4%, 47.6%, 60.0% and 68.3%; *p* < 0.001), diabetes mellitus (11.0%, 18.1%, 24.5% and 32.4%; *p* < 0.001) and heart disease (24.4%, 27.4%, 31.5% and 35.3%; *p* < 0.001) (Table 2).

### 3.3. HRQoL, Self-rated Health and Self-rated Happiness Scores by BMI Categories

After Bonferroni adjustment, the results showed that compared with normal-weight participants, overweight participants did not have significantly different HRQoL PCS, self-rated health and self-rated happiness scores (PCS: 48.2 ± 8.9 vs. 48.2 ± 8.5; self-rated health: 69.6 ± 12.3 vs. 69.8 ± 12.3; self-rated happiness: 73.9 ± 14.7 vs. 75.1 ± 13.9, respectively) scores but they had higher MCS scores (MCS: 57.8 ± 8.1 vs. 58.8 ± 7.7, *p* < 0.05). Overweight participants scored significantly higher on HRQoL MCS and self-rated happiness than did underweight participants (MCS: 58.8 ± 7.7 vs. 56.6 ± 8.3; happiness: 75.1 ± 13.9 vs. 71.8 ± 14.0, respectively, both *p* < 0.05). Obese had significantly lower PCS scores than normal weight and overweight (46.6 ± 8.8 vs. 48.2 ± 8.9 and 46.6 ± 8.8 vs. 48.2 ± 8.5, respectively, *p* < 0.05) (Table 3).

### 3.4. Unadjusted Analysis of Associations between Independent Variables and Scores of PCS, MCS, Self-Rated Health and Self-Rated Happiness

Unadjusted analysis showed that underweight and obese had significantly lower PCS scores compared with normal weight; for MCS scores, overweight and obese had significantly better scores than normal weight; for self-rated health scores, underweight had significantly lower scores than normal weight and for self-rated happiness scores, overweight had significantly better scores than normal weight. The results of these analyses are shown in Table 4. 

### 3.5. Hierarchical Regression Models for the Associations between BMI Categories and HRQoL PCS, MCS, Self-Rated Health and Self-Rated Happiness

For the PCS model, after adjusting for covariates, compared with normal-weight participants, overweight participants did not have significantly different PCS scores (B = 0.20, 95% confidence interval [CI]: −0.45 to 0.85, *p* = 0.546) but obese participants had significantly lower PCS scores (B = −0.97, 95% CI: −1.68 to −0.26, *p* = 0.007). Experiences of stroke, heart diseases, diabetes mellitus, or hypertension were negatively associated with PCS score (B = −7.07, 95% CI: −8.40 to −5.73, *p* < 0.0001; B = −2.21, 95% CI: −2.81 to −1.60, *p* < 0.0001; B = −1.22, 95% CI: −1.88 to −0.57, *p*< 0.0001; and B = −0.88, 95% CI: −1.46 to −0.30, *p* = 0.003, respectively). Participants who lived alone had significantly lower PCS scores than did those who lived with others (B = −1.45, 95% CI: −2.49 to −0.42, *p* = 0.006). Being a volunteer was positively and significantly associated with PCS score (B = 2.36, 95% CI: 1.51 to 3.21, *p* < 0.0001). Former smoker had significantly lower PCS score than nonsmoker (B = −0.96, 95% CI: −1.91 to −0.02, *p* = 0.046).

For the MCS model, after adjusting for covariates, overweight and obese participants had significantly better MCS scores than did normal-weight participants (B = 1.00, 95% CI: 0.40 to 1.61, *p =* 0.001 and B = 1.22, 95% CI: 0.60 to 1.88, *p* < 0.0001, respectively). Stroke and heart diseases were negatively and significantly associated with MCS score (B = −2.24, 95% CI: −3.48 to −0.99, *p* < 0.0001 and B = −1.28, 95% CI: −1.84 to −0.71, *p* < 0.0001, respectively).

For the self-rated health model, after adjusting for covariates, overweight participants had significantly higher health scores than did normal-weight participants (B = 1.08, 95% CI: 0.16 to 2.00, *p* = 0.022) but underweight participants had significantly lower scores than did normal-weight participants (B = −2.88, 95% CI: −4.81 to −0.95, *p* = 0.003). Stroke, heart diseases, diabetes mellitus and hypertension were negatively associated with self-rated health (B = −2.54, 95% CI: −4.44 to −0.65, *p* = 0.009; B = −4.08, 95% CI: −4.94 to −3.22, *p* < 0.0001; B = −2.33, 95% CI: −3.26 to −1.40, *p* < 0.0001 and B = −2.08, 95% CI: −2.90 to −1.30, *p* < 0.0001, respectively). Being a volunteer was positively and significantly associated with self-rated health (B = 3.38, 95% CI: 2.19 to 4.58, *p* < 0.0001). Former smoker had significantly lower self-rated health score than nonsmoker (B = −2.32, 95% CI: −3.67 to −0.99, *p* = 0.001).

For the self-rated happiness model after adjusting for covariates, compared with normal-weight participants, overweight and obese participants had significantly better scores (B = 1.55, 95% CI: 0.45 to 2.66, *p* = 0.006 and B = 1.68, 95% CI: 0.49 to 2.88, *p =* 0.006 respectively) but underweight participants had non-significantly lower scores (B = −2.30, 95% CI: −4.61 to 0.004, *p* = 0.05). Experience of stroke and heart diseases were negatively associated with self-rated happiness (B = −2.73, 95% CI: −5.01 to −0.44, *p* < 0.019 and B = −2.46, 95% CI: −3.49 to −1.43, *p* < 0.0001, respectively). Being a volunteer was positively and significantly associated with self-rated happiness (B = 4.56, 95% CI: 3.13 to 5.99, *p* < 0.0001). Former smokers had significantly lower self-rated happiness scores than nonsmokers (B = −3.12, 95% CI: −4.72 to −1.11, *p* < 0.0001) (Table 5).

## 4. Discussion

This survey study of older adults living in Yilan City showed that, after adjusting for covariates including stroke, heart diseases, diabetes mellitus, hypertension, sex, age, education level, living status, volunteering and habits of alcohol consumption and cigarette smoking, overweight participants had significantly better MCS, self-rated health and self-rated happiness scores, compared with normal-weight participants. In contrast, underweight participants scored significantly lower than did normal-weight individuals on self-rated health. Obese individuals had significantly better MCS and self-rated happiness scores but they had significantly worse PCS scores, compared with those of normal weight.

The findings that overweight individuals had better mental HRQoL and self-rated health scores than did normal-weight individuals are in line with the results of previous studies conducted in England [18] and the United States [23]. The English study used data from the 2003 Health Survey for England to analyze the association between BMI and HRQoL (evaluated by the EQ-5D), finding that the BMIs associated with the highest HRQoL scores were 26.0 kg/m^2^ for men and 24.5 kg/m^2^ for women [18]. The United States study found that, compared with normal-weight adults, older overweight adults had equally favorable quality of life levels, as evaluated by the Health Status Questionnaire-12 [23]. Another United States study of community-dwelling adults aged 35–89 years found that overweight Black Americans had better subjective health outcomes, including MCS scores, Health Utilities Index Mark 2 and Health Utilities Index Mark 3, than did Black Americans in other BMI categories [24]. Another study across South Korea, Japan, China and Taiwan found that overweight individuals in China had better self-rated health than individuals of normal weight [4]. Likewise, in the current study, we found that people who were overweight had higher happiness scores than did those of normal weight.

One possible explanation for the finding of better subjective health outcomes among overweight participants is the nature of the study setting. In this community, older adults might have needed more weight to cope with intensive farm work when they were younger, so heavier individuals may feel that they can manage their work better. This result is in line with a recent study in the United States that analyzed data from the Medical Expenditure Panel Survey 2004–2013 [40]. A second explanation for this finding is that, although overweight and obese individuals have an increased risk of metabolic syndrome, diabetes, cardiovascular diseases and certain cancers, they do not necessarily become ill. If someone has a disease, his/her perspectives and attitudes about the disease will influence the experience of illness [41]. A third explanation for our findings is that the social views of overweight/obesity and the social meanings of HRQoL, self-rated health and happiness differ across countries. Noh et al. found that people who were overweight or obese were more acceptable in China than in Korea or Japan [4]. Fourth, the cut points used for BMI in this study could be another factor related to these findings; however, our findings were similar to those of other studies, including those employing the commonly used World Health Organization classification of BMI [19,23,24] or the revised Asia-Pacific region classification [4]. Fifth, survivor effect may be another explanation. The older adults who participated in this study may be in better physical condition, which increased their interest and allowed them to meet the inclusion criteria for the study. Thus, there might be complicated mechanisms behind BMI, sex, culture, race, socioeconomic status, other unmeasured factors and subjective health outcomes and prospective studies are needed to further investigation.

The associations of BMI with PCS and self-rated health found in the present study are similar to the associations found between BMI and mortality risk among older adults in previous work. A previous meta-analysis conducted by Winter et al. found a U-shaped relationship between BMI and all-cause mortality among older adults, with an increased risk of mortality among older adults with a BMI < 23.0 kg/m^2^ or > 33.0 kg/m^2^ [38]. Flegal et al. [12] found that the relationship between BMI and mortality varied by cause of death and that being underweight was associated with an increased risk of mortality from non-cancer-related illnesses and non-cardiovascular causes. Obesity was associated with a significantly increased risk of death from cardiovascular diseases, certain cancers, diabetes, or kidney diseases. Being overweight was associated with an increased risk of death from diabetes and kidney disease; however, overweight status was associated with a decreased risk of death from other non-cancer, non-cardiovascular causes and was not associated with risk of death from cancer or cardiovascular diseases. Therefore, being overweight was associated with a significantly decreased all-cause mortality [7] The analysis in the present study show that, after controlling for comorbidities (heart disease, stroke, diabetes and hypertension), overweight individuals did not have significantly different PCS scores but better MCS, self-rated health and self-rated happiness scores, compared with normal-weight individuals. Being overweight was found to be an independent variable with a non-detrimental or positive effect on subjective health outcomes. It probably means that if older people who are overweight but do not have heart disease, stroke, diabetes or hypertension they would have better subjective health outcomes.

After controlling for covariates, we found that obese participants had worse PCS scores but better MCS and self-rated happiness scores than did normal-weight individuals. Obesity is a risk factor for developing metabolic syndrome, diabetes mellitus, cardiovascular diseases, certain cancers and disabilities, which may lead to physical function impairment and poorer physical HRQoL. However, having a disease does not necessarily equate to being ill. The Chinese idiom “Sin kuan ti pan,” which means “If someone is in a good mood, he/she might grow fat,” expresses the belief that emotional factors may have a greater influence on illness than do physical factors, such as being overweight. A recent follow-up study in China may also support this deduction, finding that urban Chinese adults who have higher levels of happiness tend to have higher BMIs [42].

Older adults who are underweight have a higher risk of sarcopenia and frailty, which are associated with greater risks of disability, morbidity and mortality [11,13,14,16,17,26,30,43]. Therefore, underweight individuals might experience more limitations in activities and daily work; this may explain why such individuals showed significantly lower scores on self-rated health and self-rated happiness, compared with their normal-weight counterparts (after controlling for covariates). Therefore, preventing older adults from becoming underweight is important, not only to maintain good HRQoL but also to decrease the risks of sarcopenia, frailty, disability, morbidity and even mortality.

This study has several limitations. First, because of the cross-sectional design, a causal relationship between BMI and subjective health outcomes could not be determined. Second, the chronic conditions included in this study were hypertension, heart disease, diabetes mellitus and stroke; other conditions such as chronic pain or psychiatric disorders, or cancer were not recorded. Third, other unmeasured biological factors, socioeconomic status and behaviors were also noted examined. But we have tried to draw the DAG to help to clarity the associations between BMI, covariates and study outcomes with careful control for possible confounding factors when available. Fourth, the study was conducted in a moderately urban community and the results might not be generalizable to older adults in all communities. Fifth, BMI is not the best method to measure adiposity accurately [41], although it is a commonly used and practical measurement for classifying participants as underweight, normal-weight, overweight, or obese in large-scale community-based studies. Despite the abovementioned limitations, the results of this community-based survey study showed reasonable 95% confidence intervals of the estimated associations between BMI category and subjective health outcomes and covariates as well which means we have little chance to miss the true associations between BMI and subjective health outcomes in this study. The results of this cross-sectional study provided information to help us think over the ideal body weight for older adult to have better subjective health outcomes, which is another important health outcome other than disease. However, further longitudinal studies should be considered to explore the mechanisms underlying the associations between BMI category and various aspects of subjective health outcomes.

## 5. Conclusions

We found that, after adjusting for covariates, overweight participants had significantly higher mental HRQoL, self-rated health and self-rated happiness scores and did not have significantly different physical HRQoL scores, compared with normal-weight participants. Underweight older people reported poorer self-rated health and obese reported poorer physical HRQoL but better mental HRQoL and self-rated happiness. For older adults, maintaining a body weight that is overweight but not obese might result in better subjective health outcomes.

## Figures and Tables

**Table 1:** Demographic Characteristics of Participants (Total *n* = 3722).

Characteristic	Total	Men	Women	*p*-Value
	*n* (%)	*n* (%)	*n* (%)	
Total	3722 (100)	1611 (43.3)	2111 (56.7)	<0.001
Age, years				
65–74	1912 (51.4)	732 (45.4)	1180 (55.9)	
75–84	1443 (38.8)	668 (41.5)	775 (36.7)	
≥85	367 (9.9)	211 (13.1)	156 (7.4)	
BMI category				0.009
Underweight	164 (4.4)	73 (4.5)	91 (4.3)	
Normal weight	1500 (40.3)	674 (41.8)	826 (39.1)	
Overweight	1154 (31.0)	517 (32.1)	637 (30.2)	
Obese	904 (24.3)	347 (21.5)	557 (26.4)	
Educational level				<0.001
Illiterate	740 (19.9)	136 (8.5)	604 (28.7)	
Literate/Elementary school	1605 (43.1)	642 (40.1)	963 (45.7)	
Junior or senior high school	958 (25.7)	512 (32.0)	446 (21.2)	
University or above	403 (10.8)	311 (19.4)	92 (4.4)	
Living status				<0.001
Living alone	276 (7.4)	82 (5.1)	194 (9.2)	
Living with others	3445 (92.0)	1529 (94.9)	1916 (90.8)	
Personal characteristics				<0.001
Smoking				
Nonsmoker	2826 (75.9)	767 (47.6)	2059 (97.5)	
Former smoker	541 (14.5)	523 (32.5)	18 (0.9)	
Current smoker	355 (9.5)	321 (19.9)	34 (1.6)	
Alcohol use				<0.001
Nondrinker	3031 (81.4)	1020 (63.3)	2011 (95.3)	
Former drinker	191 (5.1)	180 (11.2)	11 (0.5)	
Current drinker	355 (9.5)	411 (25.5)	89 (4.2)	
Volunteering				0.844
Yes	432 (11.6)	189 (11.8)	243 (11.6)	
No	3260 (87.6)	1410 (88.2)	1850 (88.4)	

BMI: Body Mass Index; *p*-values were from Chi-squared tests.

**Table 2:** Medical Conditions of Participants by Body Mass Index Category (Total *n* = 3722).

Medical Condition		BMI Category				*p*-Value
	Total *n* (%)	Underweight (*n* = 164) *n* (%)	Normal Weight (*n* = 1499) *n* (%)	Overweight (*n* = 1153) *n* (%)	Obese (*n* = 904) *n* (%)	
Hypertension						<0.001
Count	2086 (56.0)	63 (38.4)	714 (47.6)	692 (60.0)	617 (68.3)	
Diabetes mellitus						<0.001
Count	866 (23.3)	18 (11.0)	272 (18.1)	283 (24.5)	293 (32.4)	
Heart disease						<0.001
Count	1132 (30.4)	40 (24.4)	410 (27.5)	363 (31.5)	319 (35.5)	
Stroke						0.561
Count	167 (4.5)	8 (4.9)	67 (4.5)	44 (3.8)	48 (5.3)	

BMI: Body Mass Index; *p*-Value: Linear by Linear Association in Chi-squared Test.

**Table 3:** Scores of Health-related Quality of Life (HRQoL), Self-rated Health and Self-rated Happiness by Body Mass Index Category.

Subjective Health Outcomes	All Participants	BMI Category				*p*-Value for Wald Test
		Underweight	Normal Weight	Overweight	Obese	
	Mean (SD)	Mean (SD)	Mean (SD)	Mean (SD)	Mean (SD)	
HRQoL (SF-12 v2) (*n* = 3699)						
PCS	47.7 (8.9)	46.4 (10.5)	48.2 (8.9)	48.2 (8.5)	46.6 (8.8) ^a,b^	<0.001
MCS	58.3 (7.6)	56.6 (8.3) ^b,c^	57.8 (8.1) ^b,c^	58.8 (7.7) ^a,d^	58.9 (7.4) ^a,d^	<0.001
Self-rated health (*n* = 3640)	69.3 (12.3)	67.5 (13.0)	69.6 (12.3)	69.8 (12.3)	68.6 (12.0)	0.029
Self-rated happiness (*n* = 3610)	74.4 (14.3)	71.8 (14.0) ^b^	73.9 (14.7)	75.1 (13.9) ^d^	74.7 (14.1)	0.015

BMI: Body Mass Index; HRQoL: Health-Related Quality of Life; SF-12 v2: Short Form -12 version 2; PCS: Physical Component Summary; MCS: Mental Component Summary; ^a^: Significant differences compared with normal weight (*p*-Value < 0.05); ^b^: Significant differences compared with overweight (*p*-Value < 0.05); ^c^: Significant differences compared with obese (*p*-Value < 0.05); ^d^: Significant differences compared with underweight (*p*-Value < 0.05); All *p*-alues were adjusted using Bonferroni corrections.

**Table 4:** Unadjusted Analysis of Associations between Independent Variables and Scores of PCS, MCS, Self-rated Health and Self-rated Happiness.

Variable	Model for PCS			Model for MCS			Model for Self-Rated Health			Model for Self-Rated Happiness		
	(*n* = 3643)			(*n* = 3643)			(*n* = 3585)			(*n* = 3562)		
	B	95% CI	*p*-Value	B	95% CI	*p*-Value	B	95% CI	*p*-Value	B	95% CI	*p*-Value
BMI, kg/m^2^												
Underweight	−1.80	−3.23 to −0.37	0.014	−1.19	−2.46 to 0.08	0.066	−2.08	−4.08 to −0.08	0.042	−2.14	−4.48 to 0.20	0.073
Normal weight	Reference	Reference		Reference	Reference		Reference	Reference		Reference	Reference	
Overweight	− 0.05	-0.73 to 0.63	0.895	1.01	0.41 to 1.62	0.001	0.26	−0.70 to 1.22	0.593	1.22	0.10 to 2.34	0.032
Obese	−1.60	−2.33 to − 0.87	<0.0001	1.16	0.51 to 1.81	<0.0001	−0.96	−0.98 to 0.07	0.067	0.83	−0.36 to 2.03	0.173
Stroke	−7.88	−9.25 to −6.50	<0.0001	−2.28	−3.52 to −1.05	<0.0001	−3.49	−5.44 to −1.54	<0.0001	−3.84	−6.15 to −1.54	.001
Heart diseases	−3.13	−3.74 to −2.52	<0.0001	−1.31	−1.86 to −0.76	<0.0001	−5.06	−5.92 to −4.21	<0.0001	−3.25	−4.26 to −2.23	<0.0001
Diabetes	−1.99	-2.66 to -1.32	<0.0001	−0.02	0.58 to 0.62	0.943	−3.65	−4.59 to −2.71	<0.0001	−1.51	−2.62 to −0.41	0.007
Hypertension	−2.22	−2.79 to −1.64	<0.0001	0.11	−0.40 to 0.62	0.676	−3.49	−4.29 to −2.69	<0.0001	−1.80	−2.74 to −0.86	<0.0001
Male sex	0.68	0.10 to 1.25	0.021	0.83	0.32 to 1.34	0.001	1.47	0.67 to 2.28	<0.0001	0.73	−0.22 to 1.67	0.131
Age, years												
65–74	Reference	Reference		Reference	Reference		Reference	Reference		Reference	Reference	
75–84	−2.41	−3.01 to −1.81	<0.0001	−0.73	−1.27 to −0.19	0.008	−1.06	1.91 to −0.21	0.015	−1.63	−2.62 to −0.63	0.001
≥85	−4.46	−5.44 to −3.48	<0.0001	−0.67	−1.55 to 0.21	0.136	0.78	−0.62 to 2.18	0.272	−1.37	−3.01 to 0.27	0.101
Education level												
Illiterate	Reference	Reference		Reference	Reference		Reference	Reference		Reference	Reference	
Literate/elementary	2.11	1.34 to 2.87	<0.0001	0.21	−0.48 to 0.89	0.554	3.17	2.10 to 4.23	<0.0001	3.7	2.45 to 4.94	<0.0001
school												
Junior or senior high	3.57	2.73 to 4.41	<0.0001	0.48	−0.28 to 1.23	0.216	5.35	4.18 to 6.23	<0.0001	5.47	4.10 to 6.84	<0.0001
school												
University or above	3.79	2.73 to 4.86	<0.0001	1.38	0.43 to 2.33	0.004	7.1	5.62 to 8.59	<0.0001	7.88	6.14 to 9.61	<0.0001
Living alone	−1.19	−2.29 to −0.10	0.032	−0.33	−1.30 to 0.64	0.503	0.85	−0.67 to 2.37	.274	1.54	−0.24 to 3.32	0.089
Volunteer	3.49	2.61 to 4.37	<0.0001	0.53	−0.27 to 1.32	0.185	4.25	3.02 to 5.48	<0.0001	5.56	4.15 to 7.03	<0.0001
Alcohol												
Nondrinker	Reference	Reference		Reference	Reference		Reference	Reference		Reference	Reference	
Current drinker	1.96	1.13 to 2.80	<0.0001	1.64	0.90 to 2.39	<0.0001	2.2	0.85 to 3.20	0.001	2.35	0.97 to 3.72	0.001
Former drinker	−1.22	−2.52 to 0.08	0.065	0.83	−0.31 to 1.98	0.158	−0.40	−2.24 to 1.43	0.668	−0.50	−2.63 to 1.63	0.643
Smoking												
Nonsmoker	Reference	Reference		Reference	Reference		Reference	Reference		Reference	Reference	
Current smoker	0.67	−0.31 to 1.65	0.177	0.26	−0.47 to 0.93	0.485	−0.77	−0.61 to 2.14	0.276	−0.96	−2.57 to 0.64	0.24
Former smoker	−1.10	−1.69 to −0.29	0.008	1.09	0.23 to 1.96	0.014	−1.77	−2.92 to −0.62	0.002	−2.35	−3.68 to −1.01	0.001

BMI: Body Mass Index; PCS: Physical Component Summary; MCS: Mental Component Summary; Results were from unadjusted linear regression models and *p*-Values had been adjusted using the Bonferroni correction.

**Table 5:** Associated Factors of PCS, MCS, Self-rated Health and Self-rated Happiness Scores by Hierarchical Regression.

Variable	Model for PCS			Model for MCS			Model for Self-Rated Health			Model for Self-Rated Happiness		
	(*n* = 3643)			(*n* = 3643)			(*n* = 3585)			(*n* = 3562)		
	B	95% CI	*p*-Value	B	95% CI	*p*-Value	B	95% CI	*p*-Value	B	95% CI	*p*-Value
BMI, kg/m^2^												
Underweight	−1.31	−2.67 to 0.05	0.059	−1.02	−2.29 to 0.25	0.116	−2.88	−4.81 to −0.95	0.003	−2.30	−4.61 to 0.004	0.05
Normal weight	Reference	Reference		Reference	Reference		Reference	Reference		Reference	Reference	
Overweight	0.20	− 0.45 to 0.85	0.546	1.00	0.40 to 1.61	0.001	1.08	0.16 to 2.00	0.022	1.55	0.45 to 2.66	0.006
Obese	−0.97	−1.68 to −0.26	0.007	1.22	0.60 to 1.88	<0.0001	−0.69	−0.32 to 1.69	0.179	1.68	0.49 to 2.88	0.006
Stroke	−7.07	−8.40 to −5.73	<0.0001	−2.24	−3.48 to −0.99	<0.0001	−2.5	−4.44 to −0.65	0.009	−2.73	−5.01 to −0.44	0.019
Heart diseases	−2.21	−2.81 to −1.60	<0.0001	−1.28	−1.84 to −0.71	<0.0001	−4.08	−4.94 to −3.22	<0.0001	−2.46	−3.49 to −1.43	<0.0001
Diabetes	−1.22	−1.88 to −0.57	<0.0001	0.04	−0.58 to 0.65	0.91	−2.33	−3.26 to −1.40	<0.0001	−0.82	−1.93 to 0.29	0.148
Hypertension	−0.88	−1.46 to −0.30	0.003	0.32	−0.22 to 0.86	0.241	−2.08	−2.90 to −1.30	<0.0001	−0.95	−1.92 to −0.03	0.058
Male sex	0.95	0.20 to 1.70	0.013	0.51	− 0.19 to 1.20	0.155	0.75	− 0.32 to 1.81	0.168	0.11	−1.16 to 1.38	0.864
Age, years												
65–74	Reference	Reference		Reference	Reference		Reference	Reference		Reference	Reference	
75–84	−1.64	−2.23 to −1.05	<0.0001	−0.53	−1.09 to 0.02	0.058	0.52	−0.32 to 1.35	0.228	−0.03	−1.04 to 0.97	0.947
≥ 85	−3.79	−4.76 to −2.82	<0.0001	−0.20	−1.10 to 0.70	0.664	2.27	0.27 to 3.65	0.001	0.44	−1.20 to 2.09	0.598
Education level												
Illiterate	Reference	Reference		Reference	Reference		Reference	Reference		Reference	Reference	
Literate/elementary	1.63	0.88 to 2.37	<0.0001	−0.000087	−0.69 to 0.69	1.00	2.77	1.71 to 3.83	<0.0001	3.57	2.31 to 4.83	<0.0001
school												
Junior or senior high	2.52	1.67 to 3.37	<0.0001	0.18	−0.61 to 0.97	0.659	4.55	3.34 to 5.75	<0.0001	5.07	3.62 to 6.51	<0.0001
school												
University or above	2.27	1.17 to 3.38	<0.0001	0.93	−0.10 to 1.96	0.077	5.94	4.74 to 7.51	<0.0001	7.36	5.49 to 9.23	<0.0001
Living alone	−1.45	−2.49 to −0.42	0.006	−0.47	−1.44 to 0.49	0.338	0.6	−0.87 to 2.05	0.427	1.28	−0.46 to 3.03	0.150
Volunteer	2.36	1.51 to 3.21	<0.0001	0.23	−0.56 to 1.02	0.564	3.38	2.19 to 4.58	<0.0001	4.56	3.13 to 5.99	<0.0001
Alcohol												
Nondrinker	Reference	Reference		Reference	Reference		Reference	Reference		Reference	Reference	
Current drinker	0.99	0.13 to 1.84	0.023	1.09	0.30 to 1.89	0.007	0.96	− 0.26 to 2.18	0.122	1.98	0.52 to 3.43	0.008
Former drinker	−0.67	−1.99 to 0.64	0.315	0.65	−0.58 to 1.88	0.299	−0.13	−2.01 to 1.75	0.894	0.85	− 1.38 to 3.09	0.455
Smoking												
Nonsmoker	Reference	Reference		Reference	Reference		Reference	Reference		Reference	Reference	
Current smoker	−0.34	−1.37 to 0.70	0.525	0.57	−0.40 to 1.54	0.246	-0.3	−0.78 to 1.18	0.692	−1.61	−3.37 to 0.17	0.074
Former smoker	−0.96	−1.91 to −0.02	0.046	−0.09	−0.97 to 0.80	0.839	−2.32	−3.67 to −0.99	0.001	−3.12	−4.72 to −1.11	<0.0001
Adjusted R^2^	0.118			0.02			0.096			0.058		

BMI: Body Mass Index; PCS: Physical Component Summary; MCS: Mental Component Summary; Results were from adjusted linear regression models and *p*-values had been adjusted using the Bonferroni correction.
